# Cytolytic DNA vaccine encoding lytic perforin augments the maturation of- and antigen presentation by- dendritic cells in a time-dependent manner

**DOI:** 10.1038/s41598-017-08063-1

**Published:** 2017-08-17

**Authors:** Danushka K. Wijesundara, Wenbo Yu, Ben J. C. Quah, Preethi Eldi, John D. Hayball, Kerrilyn R. Diener, Ilia Voskoboinik, Eric J. Gowans, Branka Grubor-Bauk

**Affiliations:** 1 Virology Laboratory, The Basil Hetzel Institute for Translational Health Research, The University of Adelaide, Adelaide, South Australia Australia; 2 Translational Oncology Laboratory, Centre for Cancer Biology, Adelaide, South Australia Australia; 3 Cancer & Vascular Biology Laboratory, The John Curtin School of Medical Research, Canberra, The Australian Capital Territory Australia; 4 Experimental Therapeutics Laboratory, Sansom Institute for Health Research, Adelaide, South Australia Australia; 5 School of Pharmacy and Medical Sciences, University of South Australia, Adelaide, South Australia Australia; 6 Robinson Research Institute and Adelaide Medical School, University of Adelaide, Adelaide, South Australia Australia; 7 Killer Cell Biology Laboratory, Peter MacCallum Cancer Centre, Melbourne, Victoria, Australia

## Abstract

The use of cost-effective vaccines capable of inducing robust CD8^+^ T cell immunity will contribute significantly towards the elimination of persistent viral infections and cancers worldwide. We have previously reported that a cytolytic DNA vaccine encoding an immunogen and a truncated mouse perforin (PRF) protein significantly augments anti-viral T cell (including CD8^+^ T cell) immunity. Thus, the current study investigated whether this vaccine enhances activation of dendritic cells (DCs) resulting in greater priming of CD8^+^ T cell immunity. *In vitro* data showed that transfection of HEK293T cells with the cytolytic DNA resulted in the release of lactate dehydrogenase, indicative of necrotic/lytic cell death. *In vitro* exposure of this lytic cell debris to purified DCs from naïve C57BL/6 mice resulted in maturation of DCs as determined by up-regulation of CD80/CD86. Using activation/proliferation of adoptively transferred OT-I CD8^+^ T cells to measure antigen presentation by DCs *in vivo*, it was determined that cytolytic DNA immunisation resulted in a time-dependent increase in the proliferation of OT-I CD8^+^ T cells compared to canonical DNA immunisation. Overall, the data suggest that the cytolytic DNA vaccine increases the activity of DCs which has important implications for the design of DNA vaccines to improve their translational prospects.

## Introduction

DNA is an ideal platform to develop global vaccines because it is safe, cheap to manufacture rapidly and stable even in ambient temperatures. Furthermore, a landmark phase IIb clinical trial demonstrated that electroporation of a DNA vaccine (VGX-3100) was capable of eliciting immunity to clear human papillomavirus (HPV) and completely regress HPV-induced cervical intraepithelial neoplasia in humans^1^. Although the protective mechanism of this vaccine is still not clear, it is likely that cell death and inflammation resulting from electroporation at the vaccination site are contributing factors to its efficacy^2^.

Several studies from our laboratory suggest that DNA vaccines designed to induce necrosis of vaccine-targeted cells elicit greater T cell immunity and protection than canonical DNA vaccines following intradermal (ID) immunisation^3–6^. In these studies, expression of the immunogen was driven by the cytomegalovirus (CMV) promoter while the simian virus 40 (SV40) promoter drove expression of a cytolytic protein, truncated mouse perforin (PRF). The CMV promoter is ∼10-fold more efficient in driving gene expression than the SV40 promoter^3^ and the encoded PRF contains a 12 amino acid deletion (12del PRF) at the C-terminus, resulting in cell death after expression^7^. This bicistronic plasmid design allows the immunogen to accumulate significantly within vaccine-targeted cells before necrosis occurs due to 12del PRF expression. Necrotic cell death results in release of damage associated molecular patterns (DAMPs) which act as natural adjuvants and result in inflammation and activation of antigen presenting cells (APCs)^8^. Dendritic cells (DCs) are APCs that are pivotal to initiate CD8^+^ T cell immunity and are the most efficient APC at cross-presenting antigens including antigens derived from dead cells^9^. Consequently, in this study, we investigated the hypothesis that the cytolytic DNA vaccine will activate DCs to cross-present antigens to CD8^+^ T cells more efficiently than canonical DNA vaccines which are non-cytolytic.

## Results and Discussion

**Figure 1:**
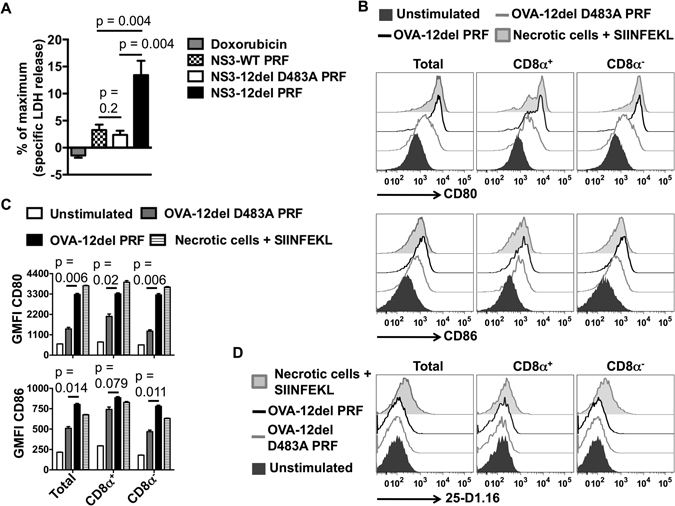
Lytic cell debris generated following cytolytic DNA expression can activate DCs. (**A**) HEK293T cells were transfected for 48 hours with 200 ng of DNA or treated for 24 hours with 2 μM doxorubicin prior to measuring LDH release into the culture supernatants. Data show the mean (n = 6) + SEM of specific LDH release from two independent experiments. (**B**) Magnetically enriched CD11c^+^ cells from naïve C57BL//6 mice (n = 3) were exposed to transfected HEK293T cells or heat-induced necrotic cells in the presence of 0.1 μg/ml SIINFEKL peptide for 8 hours prior to flow cytometric analysis as described in the methods. Representative histogram plots show CD80 and CD86 expression on gated CD11c^+^ I-Ab^+^ (total), CD8α^+^ CD11c^+^ I-Ab^+^ and CD8α^−^ CD11c^+^ I-Ab^+^ cells. Similar results were observed in two independent experiments. (**C**) Mean (n = 3) + SEM for the data described in B. A paired t-test was used to determine the statistical significance of the data. (**D**) Representative histogram plots showing SIINFEKL-H2-K^b^ (25-D1.16) expression on gated DC populations for the experiment described in B.

DCs exposed to necrotic or lytic cell environments mature and cross-present antigens to CD8^+^ T cells more efficiently than DCs exposed to healthy or apoptotic cells^10–12^ and the release of lactate dehydrogenase (LDH) from cells is indicative of lytic/necrotic cell death^13^. Consequently, to investigate whether expression of the truncated mouse perforin, 12del PRF, encoded by the bicistronic DNA vaccine resulted in lytic cell death, the release of LDH from transfected HEK293T cells was measured (Fig. 1A). In this experiment, the bicistronic DNA encoded the non-structural protein 3 (NS3) from hepatitis C virus as the immunogen. A mutant form of 12del PRF with a D483A mutation which renders PRF non-cytolytic^7, 14^ served as a control for these experiments. Transfection of HEK293T cells with the bicistronic DNA encoding 12del PRF, but not wild-type (WT) PRF, significantly increased LDH release from transfected cells compared to the control (NS3-12del D483A PRF) (Fig. 1A). In contrast, treatment of HEK293T cells with a pro-apoptotic drug, doxorubicin, failed to result in LDH release (Fig. 1A). Thus, only expression of 12del PRF from the bicistronic DNA vaccine resulted in significant necrotic cell death. These results are consistent with previous reports demonstrating the lytic activity of 12del PRF^5, 7^ and with the general understanding that apoptosis which fails to result in the release of DAMPs is non-inflammatory whereas necrosis not only results in the release of DAMPs, but is inflammatory leading to increased immunogenicity^8^.

Next, to examine if DCs are activated following exposure of lytic cell debris resulting from 12del PRF expression, HEK293T cells were transfected with cytolytic- or control- DNA encoding the model antigen ovalbumin (OVA). These cells were then exposed to purified DCs from naïve C57BL/6 mice. This experiment showed that DCs (gated based on the expression of CD11c and Major Histocompatibility Complex Class (MHC-C) II (CD11c^+^ I-Ab^+^)) including the cross-presenting CD8α^+^ subset, which were exposed to HEK293T cells transfected with the cytolytic DNA expressed higher levels of the costimulatory molecules CD80 and CD86, indicative of maturation, compared to DCs exposed to HEK293T cells transfected with control DNA (Fig. 1B and C). However, expression of the immunodominant peptide, SIINFEKL-H2-K^b^, was not detected in these DC-HEK293T co-cultures (Fig. 1D). This was not surprising because the 25-D1.16 antibody fails to detect low levels of SIINFEKL-H2-K^b^ expression and is the only antibody available to quantify this peptide- MHC-CI complex on murine cells^9^. Staining for SIINFEKL-H2-K^b^ was observed only for DC subsets analysed in co-cultures involving heat-treated necrotic cells (as described in Materials and Methods) in the presence of 0.1 μg/ml SIINFEKL peptide (positive control) (Fig. 1D). Nevertheless, the *in vitro* data (Fig. 1B,C) suggest that exposure of lytic cell debris resulting from cytolytic DNA expression can trigger maturation of DCs.

**Figure 2:**
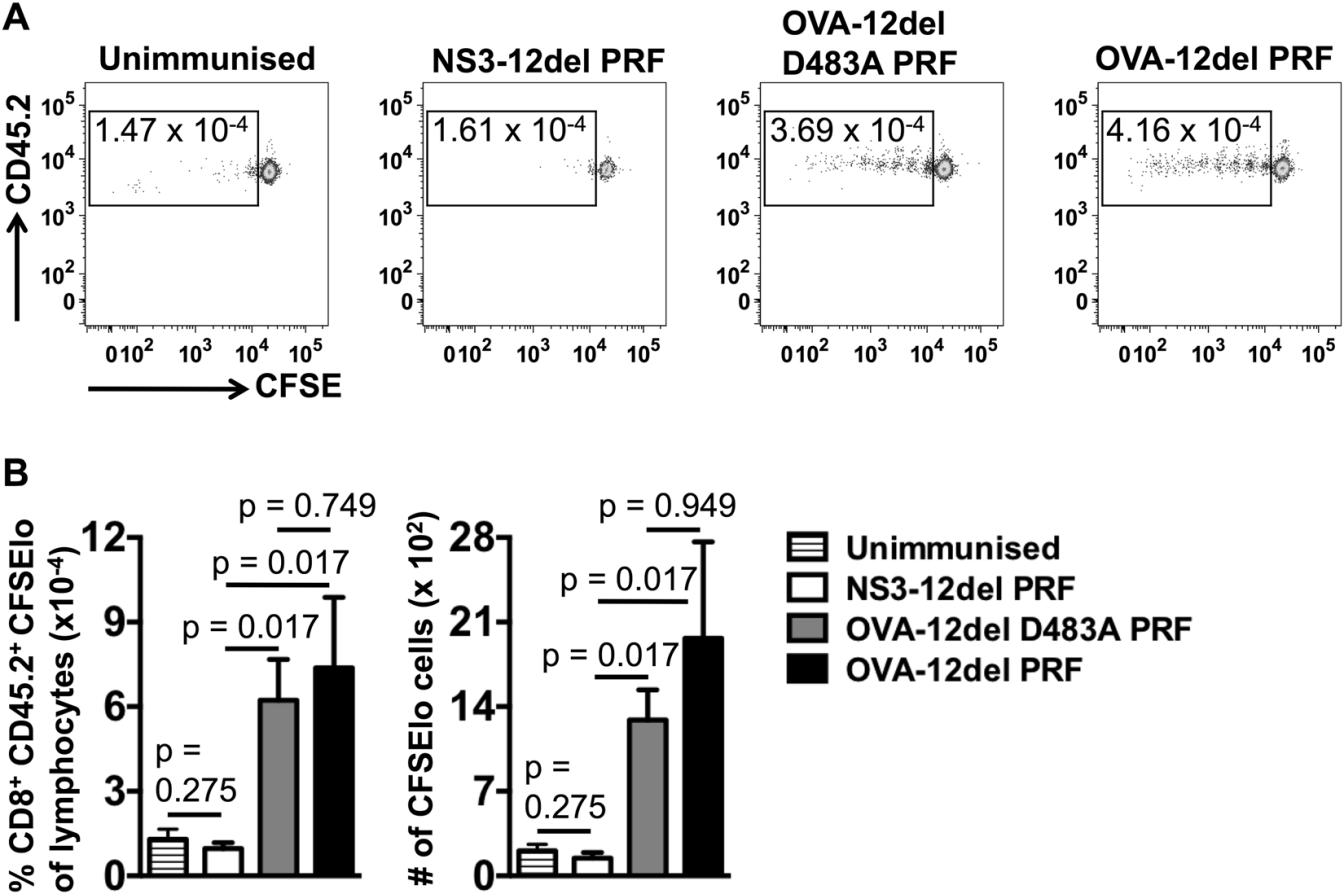
The effect of cytolytic DNA immunisation on early phases of antigen presentation to CD8^+^ T cells *in vivo*. (**A**) C57BL/6.SJL (CD45.1^+^, CD45.2^−^) recipient mice were immunised via the ID route with 100 μg of NS3-12del PRF (n = 3), OVA-12del D483A PRF (n = 7), OVA-12del PRF (n = 7) or were unimmunised (n = 3). Four days later, 5 × 10^5^ C57BL/6 Rag^−/−^ OT-I cells (CD45.2^+^) labelled with 10 μM CFSE were adoptively transferred (i.v.) into each recipient mouse and proliferation of transferred CD8^+^ cells in the CLN was monitored 4 days later. Representative dot plots obtained from CLN samples and gated on CD8^+^ CD45.2^+^ cells are shown. The numbers in the gates indicate the percentage of lymphocytes that are single cells (doublet discriminated), CD8^+^ CD45.2^+^ with reduced levels of CFSE. (**B**) Mean + SEM of the percentages indicated in A (left panel) and the mean + SEM of the absolute number of CD8^+^ CD45.2^+^ CFSElo cells (right panel). The results are representative of 2 independent experiments. Kruskal-Wallis H test was used to determine the statistical significance of the data.

Proliferation of adoptively transferred carboxyfluorescein succinimidyl ester (CFSE)-labelled OT-I CD8^+^ T cells which present OVA epitopes, e.g. SIINFEKL, in recipient mice in the context of MHC-CI is commonly used to quantify antigen presentation by DC *in vivo* ^15^. Thus, this method was used to determine whether cytolytic DNA immunisation could augment antigen presentation by DCs to CD8^+^ T cells *in vivo* (Fig. 2). The data show that immunisation with cytolytic DNA failed to increase the number of proliferating C57BL/6 Rag ^−/−^ OT-I CD8^+^ T cells in the draining cervical lymph nodes (CLN) compared to the control when early phases of antigen presentation (days 4-8) were examined (Fig. 2). Similar results were generated when C57BL/6 OT-I CD8^+^ T cells (as opposed to C57BL/6 Rag^−/−^ OT-I cells (CD45.2^+^)) were used for adoptive transfer and proliferation of these cells was examined 0–7 days following DNA immunisation (data not shown). However, we have demonstrated previously that cell death *in vivo* was detected 14 days after ID immunisation with cytolytic DNA^3^ and antigen presentation following ID DNA immunisation can be detected for at least 21 days post-immunisation^16^.

**Figure 3:**
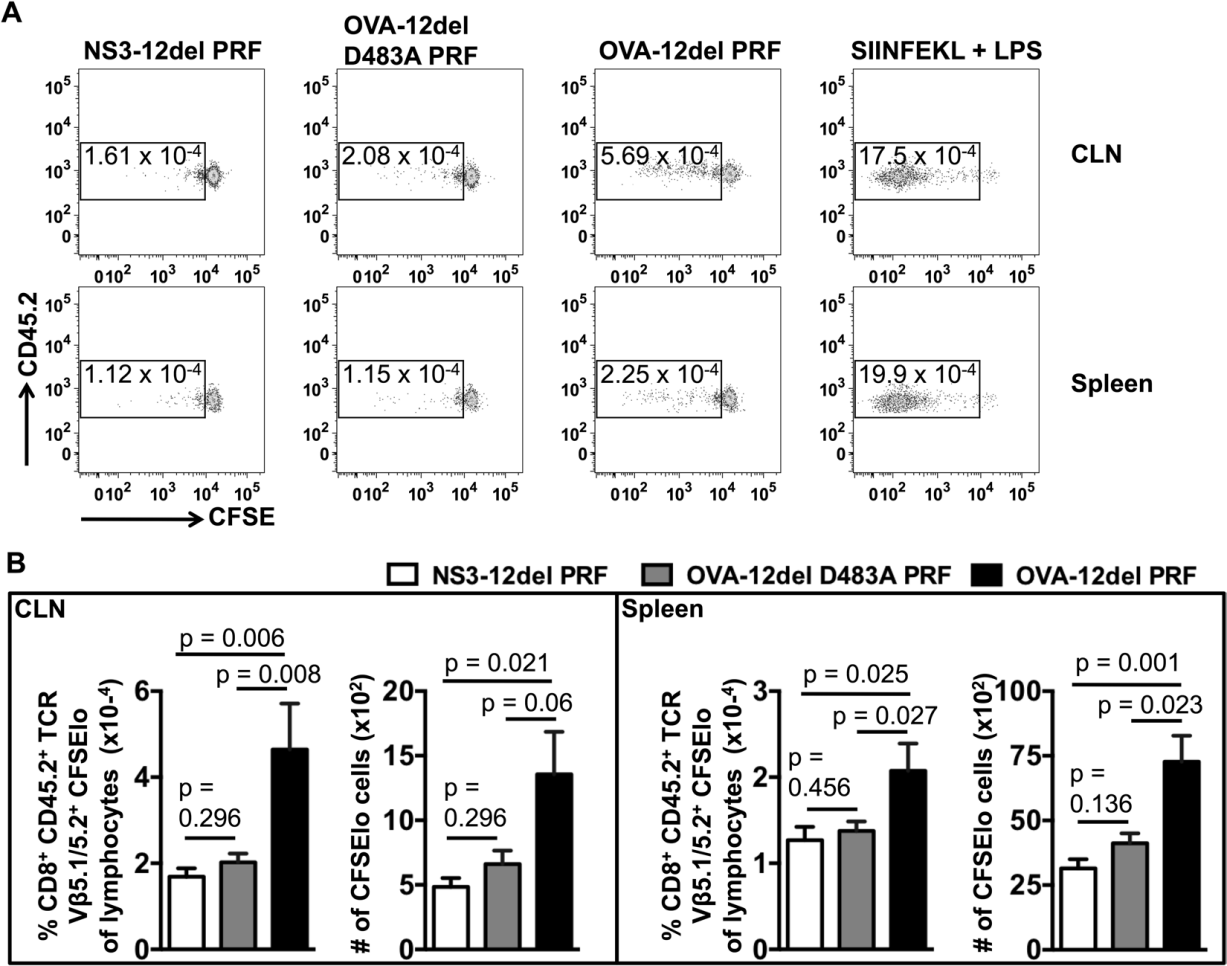
Cytolytic DNA immunisation enhances antigen presentation to CD8^+^ T cells in a time-dependent manner *in vivo*. (**A**) C57BL/6.SJL mice were immunised via the ID route with 100 μg of NS3-12del PRF (n = 7), OVA-12del D483A PRF (n = 14) or OVA-12del PRF (n = 14) prior to i.v. transfer of 30 μM CFSE-labelled C57BL/6 OT-I cells (5 × 10^6^ cells/recipient) 7 days later. 15 days after the i.v. transfer, proliferation of OT-I CD8^+^ cells was measured in the CLN and spleens of recipient mice. As a positive control, one day after the adoptive transfer of OT-I cells into naïve recipient mice (n = 2), 20 μg SIINFEKL peptide + 5 μg of LPS were injected ID per mouse prior to analysis 14 days later. Representative dot plots gated on CD8^+^ CD45.2^+^ T cell receptor (TCR) Vβ5.1/5.2^+^ are shown. The numbers in the gates indicate the percentage of lymphocytes that are single cells (doublet discriminated), CD8^+^ CD45.2^+^ TCR Vβ5.1/5.2^+^ with reduced levels of CFSE. (**B**) Mean + SEM of the percentages indicated in A (left panel) and the mean + SEM of the absolute number of CD8^+^ CD45.2^+^ TCR Vβ5.1/5.2^+^ CFSElo cells (right panel). Pooled data from two independent experiments are shown. The Kruskal-Wallis H test was used to determine the statistical significance of the data.

Consequently, we examined antigen presentation occurring from 7–22 days following ID immunisation with cytolytic or control DNA. As a positive control, one day after the adoptive transfer of OT-I cells into naïve recipient mice (n = 2), 20 μg SIINFEKL peptide +5 μg of LPS were injected ID per mouse prior to analysis 14 days later. LPS promotes maturation of antigen presenting cells and SIINFEKL injection is commonly used in adoptive transfer experiments to ensure that OT-I CD8^+^ T cells in recipients retain the capacity to proliferate and to delineate proliferating cells during flow cytometric analysis^17^. The results of these experiments show that cytolytic DNA immunisation increased the number of proliferating OT-I CD8^+^ T cells (CD45.2^+^) by ∼2-fold in the CLN as well as the spleen compared to the 12del D483A PRF control (Fig. 3). These findings are consistent with our previous report which demonstrated that a higher proportion of mature cross-presenting (i.e. CD8α^+^ CD80^+^) DCs emerge in the CLN 14 days following ID immunisation with cytolytic DNA compared to canonical DNA^3^. Furthermore, the increase in antigen presentation due to cytolytic DNA vaccination is consistent with our earlier reports which demonstrated that cytolytic DNA vaccination greatly enhances anti-viral effector CD8^+^ T cell immunity^3–6^. Thus, the data suggest that cytolytic DNA more efficiently activates DCs to present vaccine-encoded antigens to CD8^+^ T cells *in vivo*, compared to canonical DNA vaccination.

**Figure 4:**
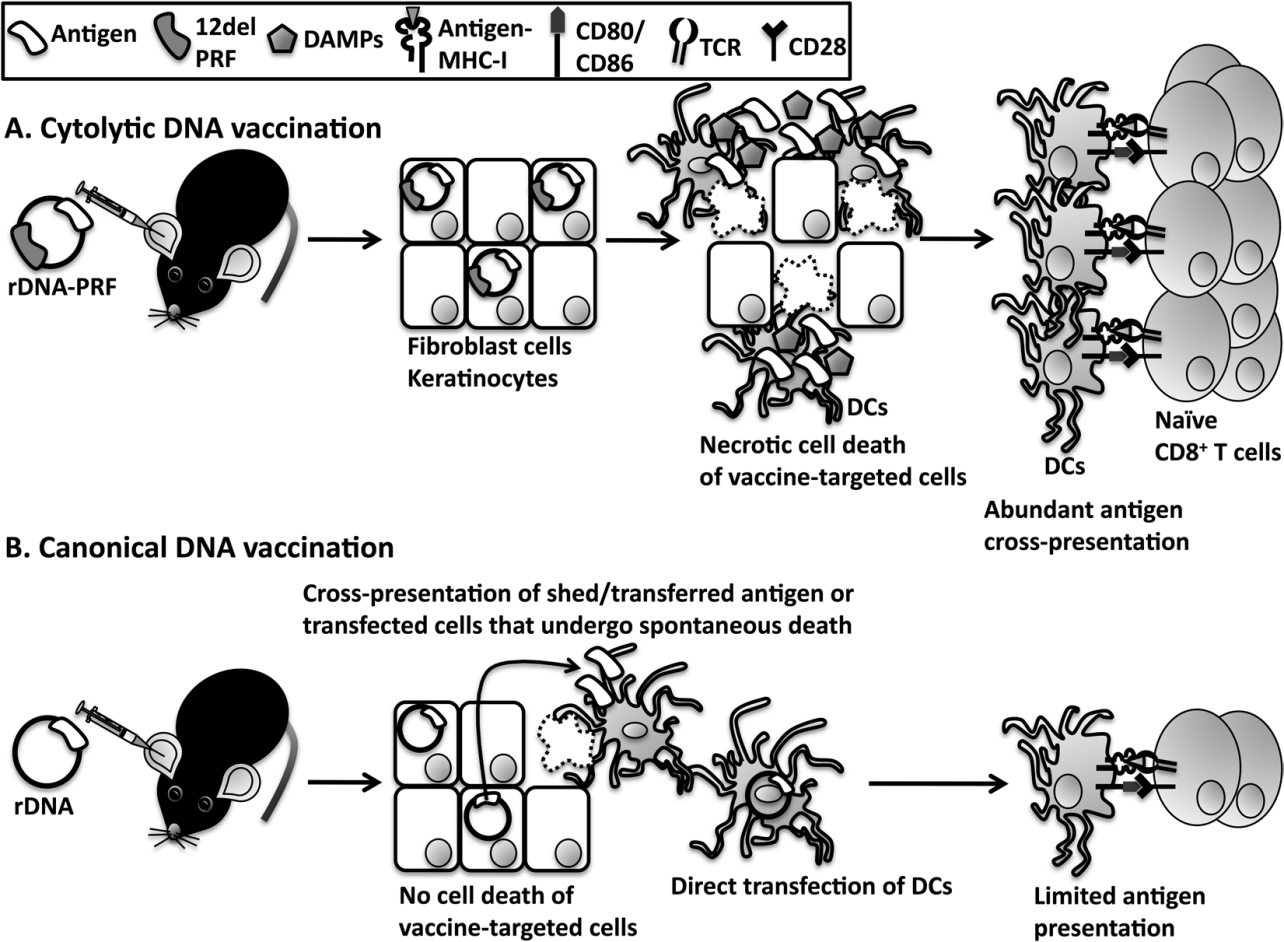
Proposed mechanism of the adjuvant activity of the cytolytic DNA vaccine. (**A**) It is favourable to deliver DNA vaccines into the dermis because it is rich in DCs, but the vast majority of the dermis is composed of fibroblasts and keratinocytes making them the main targets for DNA uptake. Following expression, 12del PRF expression in cytolytic DNA transfected cells will cause necrotic cell death leading to sterile inflammation, and exposure of intrinsic cell DAMPs and vaccine antigens to DCs. Dermal DCs, including those recruited from other anatomical sites due to sterile inflammation, will endocytose the antigen and be exposed to DAMPs resulting in maturation (up-regulation of CD80/CD86 expression). Thus DCs, the most efficient antigen cross-presenting cells *in vivo*, will in this instance efficiently prime naïve CD8^+^ T cells. This is a time-dependent process and likely depends on the accumulation of antigen and DAMPs at the dermis due to prolonged expression of cytolytic DNA in fibroblasts and keratinocytes. (**B**) Conversely, canonical DNA vaccines are unlikely to result in significant cell death and mainly rely on directly targeting DCs in the dermis and to some extent cross-presentation of transfected dead cells and transferred/shed antigens. Hence, canonical DNA vaccination is less efficient in priming naïve CD8^+^ T cells than cytolytic DNA vaccination.

DCs are crucial to initiate CD8^+^ T cell immunity, but, being a rare cell population, direct targeting of DCs is difficult unless proteins/antibodies recognizing cell surface molecules (e.g. DEC205) are tagged on the DNA or lipid molecules (e.g. liposomes) are used to encapsulate DNA^18^. However, the use of protein or antibody tags increases the manufacturing and storage costs of the vaccine, and despite the potential of these strategies, none has resulted in widespread use^2^. Immunity against the protein tags can also develop resulting in reduced efficacy of the vaccine following booster vaccinations^6^. Consequently, alternative strategies to target DNA-encoded antigens to DCs are required and the findings from the current study suggest that cytolytic DNA encoding 12del PRF can be used to deliver vaccine-encoded antigens effectively to DCs in an indirect manner and enhance antigen presentation to naïve CD8^+^ T cells in a time-dependent manner (Fig. 4).

Following ID delivery of canonical DNA, antigen delivery to DCs is crucial to prime naïve CD8^+^ T cells; this may occur after direct transfection of DCs although antigen transfer/shedding from transfected fibroblasts/keratinocytes or DCs uptake of antigen derived from dead cells may also contribute to some degree^19–21^ (Fig. 4B). However, the vast majority of the cells in the dermis are fibroblasts and keratinocytes, and consequently, the bulk of the DNA delivered to this site will likely transfect these cells (Fig. 4). Consequently, cross-presentation is necessary to efficiently prime naïve CD8^+^ T cells and it is most likely that this mechanism of antigen presentation is exploited more effectively following cytolytic DNA vaccination compared to canonical DNA vaccination (Fig. 4). A caveat for the model described in Fig. 4 is that it is based on ID delivery of canonical or cytolytic DNA into the ear pinnae. It is possible that the method of antigen presentation to CD8^+^ T cells will vary depending on the route or mode of delivery of DNA^22–25^.

It is unclear which DC subsets cross-present antigen to CD8^+^ T cells following cytolytic DNA immunisation, but based on previous reports it is unlikely that Langerhan cells are required for cross-presentation^26^. It is likely that langerin^+^ dermal DCs^27^, CD8α^+^ and/or CD103^+^ DC subsets^28, 29^ are involved, but this requires further investigation.

DNA vaccines have historically failed to show efficacy in large animal models and humans owing mainly to poor immunogenicity and delivery of DNA into cells. The recent success of using DNA vaccines to treat cervical cancer patients^1, 30^ has reinvigorated the DNA vaccine research field. Furthermore, it is likely that a DNA vaccine will also emerge to protect against Zika virus infection in humans given that a plasmid DNA vaccine conferred protection in mice and macaques against Zika virus challenge^31, 32^. The cytolytic DNA vaccine encoding 12del PRF is also more immunogenic than canonical DNA vaccines in outbred pigs^5^, a recognised large animal in which to test the immunogenicity of experimental vaccines^33^. Consequently, these findings highlight exciting translational prospects for the cytolytic DNA to be used as a platform for designing vaccines against infectious diseases and cancer.

## Materials and Methods

### Mice

All experiments were approved and performed in accordance with the guidelines and regulations of the University of Adelaide and the SA Pathology animal ethics committees. C57BL/6 and C57BL/6.SJL mice were of the 6J substrain and purchased from the Australian Resource Centre at Perth. C57BL/6 OT-I mice were purchased from the Australian Phenomics Facility at Canberra. Rag^−/−^ C57BL/6 OT-I mice were bred in specific pathogen free conditions at the Sansom Institute for Health Research, Adelaide. All mice were housed in individually ventilated HEPA-filtered cages at the Queen Elizabeth Hospital, Woodville.

### DNA preparation and immunisations

**Figure 5:**
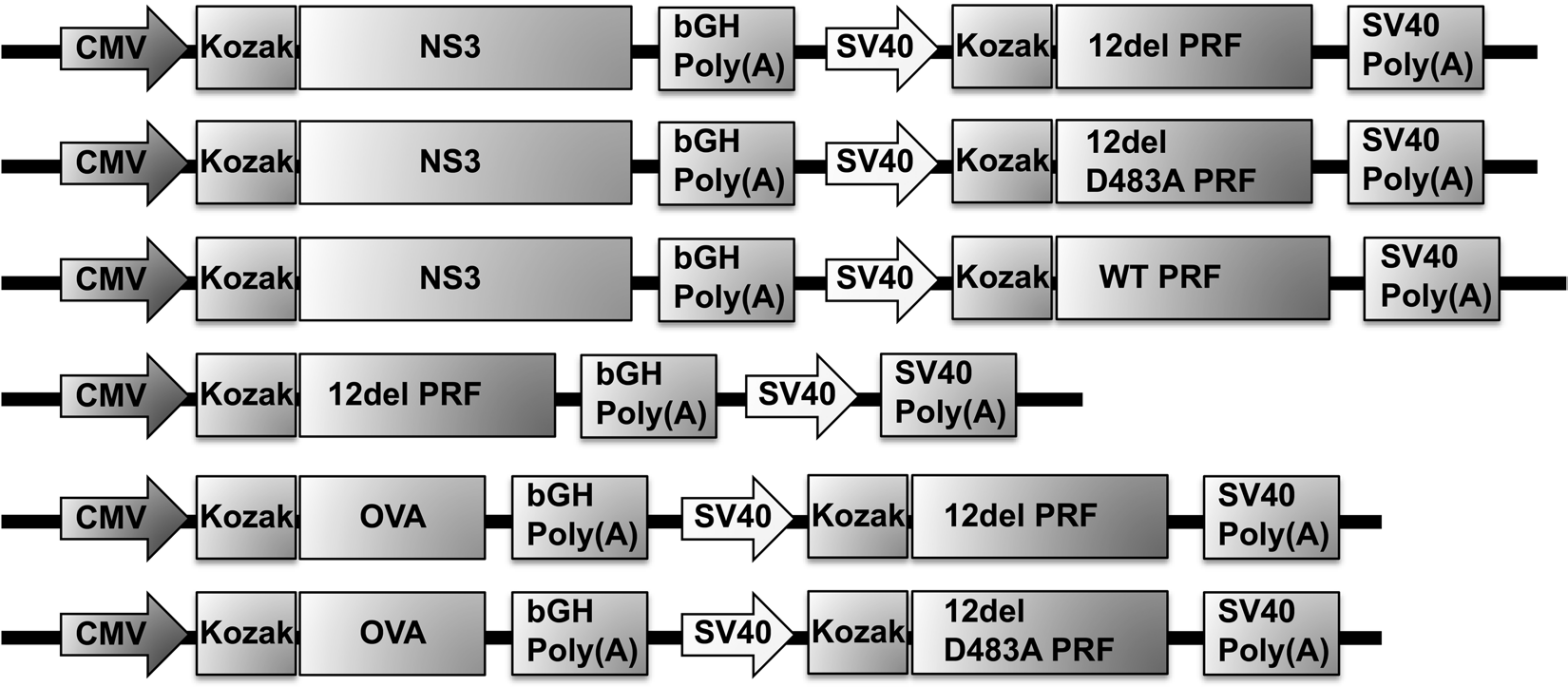
Schematic of the plasmid DNA constructs used in the study.

The cytolytic DNA was constructed using the pVAX plasmid (Life Technologies) as described previously^3^. The genes for *Gallus gallus* ovalbumin (OVA) or codon-optimised NS3^4^ were inserted downstream of the CMV promoter, and those for 12del PRF, 12del D483A PRF or WT PRF inserted downstream of the SV40 promoter. The different PRF sequences used are described in Brennan *et al* ^7^. All DNA vaccines were prepared using a well-established alkaline lysis method^34^ and endotoxins removed with an Endotoxin Removal Solution (Sigma-Aldrich) following the manufacturer’s guidelines. A schematic diagram of the plasmid DNA constructs used in this study is shown in Fig. 5.

Aged matched (6–8 weeks old during initial vaccination) mice were under isofluorane anesthesia during ID DNA immunisations into the ear pinnae using a 29 G needle and syringe. Each mouse received 100 µg of endotoxin-free DNA in phosphate buffer saline (PBS) (50 µg in 10 µl/ear). In the experiment described in Fig. 3, a proportion of the mice were immunised via the ID route with 20 μg SIINFEKL peptide +5 μg of lipopolysaccharide (LPS) in PBS (Sigma-Aldrich) as a positive control.

### LDH cytotoxicity assay

HEK293T cells were transfected with 200 ng of DNA in 96-well flat-bottom plates and LDH activity in the culture supernatant measured 48 hours later using the manufacturer’s protocol (Thermo Scientific Pierce) as we described^5^. Eight hours after transfection the Dulbecco’s Modified Eagle Medium (DMEM; Life Technologies) + 10% heat inactivated Fetal Bovine Serum (FBS) which was used as cell culture medium during transfection was replaced with DMEM + 2% FBS. To induce apoptosis of HEK293T cells, cells were treated with 2 μM doxorubicin for 24 hours as described^5^. The percentage of maximum (specific LDH release) was calculated according to the manufacturer’s protocol (Thermo Scientific Pierce).

### Adoptive transfer

Pooled splenocytes and lymph node cells from Rag^−/−^ C57BL/6 OT-I or C57BL/6 OT-I mice were labelled with 10 or 30 μM CFSE (Molecular Probes) respectively as demonstrated^17^. After washing, red blood cells (RBC) were depleted in RBC lysis buffer (155 mM NH_4_Cl + 0.01 M Tris-HCl in Milli-Q water, pH 7.65), the labelled cells resuspended in PBS and injected intravenously (i.v.) into the lateral tail vein of C57BL/6.SJL vaccinated mice.

### DC-HEK293T cell coculture

DCs were purified using splenocytes from naïve C57BL/6 mice using CD11c magnetic MicroBeads according to the manufacturer’s protocol (Miltenyi Biotec). Prior to CD11c enrichment, the spleens were digested for 45 mins at room temperature using 100 mg/ml collagenase type 3 (Worthington) + 10 mg/ml DNase I (Sigma-Aldrich) in Roswell Park Memorial Institute Medium (RPMI; Life Technologies) + 2% FBS.

HEK293T cells from 3 wells transfected with DNA as described above were cocultured for 8 hours at 37 °C + 5% CO_2_ with 1 × 10^5^ purified DCs. As a positive control, naïve HEK293T cells (cultured for 48 hours) were incubated at 56 °C for 45 minutes to induce necrosis and cultured with 1 × 10^5^ purified DCs in the presence of 0.1 μg/ml of SIINFEKL peptide. Following the coculture, the DCs were stained with antibodies that react against SIINFEKL-H-2K^b^ (25-D1.16, BioLegend), CD80 (eBioscience) and CD86 (BioLegend) to quantify antigen presentation (i.e. with 25-D1.16) and maturation (i.e. with anti-CD80 and anti-CD86) of the DCs using flow cytometric analysis. The SIINFEKL peptide used in this study was a gift from Dr. Charani Ranasinghe, The John Curtin School of Medical Research, Canberra.

### Flow cytometry

Cell surface and intracellular staining was performed similar to that described in Holmes *et al*.^35^. Antibodies against mouse antigens CD8α, T cell receptor (TCR) Vβ5.1/5.2, CD80 and CD11c were purchased from eBioscience. Antibodies against mouse antigen I-Ab (MHC-CII) was from BD Pharmingen. All the other antibodies were purchased from BioLegend.

### Statistical analysis

All graphs were constructed using GraphPad Prism 6 software and the p values of the data were calculated using a paired t-test or Kruskal-Wallis H test of the IBM SPSS Statistics software (version 24).

## References

[1] Trimble CL (2015). Safety, efficacy, and immunogenicity of VGX-3100, a therapeutic synthetic DNA vaccine targeting human papillomavirus 16 and 18 E6 and E7 proteins for cervical intraepithelial neoplasia 2/3: a randomised, double-blind, placebo-controlled phase 2b trial. Lancet.

[2] Jorritsma SH, Gowans EJ, Grubor-Bauk B, Wijesundara DK (2016). Delivery methods to increase cellular uptake and immunogenicity of DNA vaccines. Vaccine.

[3] Gargett T (2014). Induction of antigen-positive cell death by the expression of perforin, but not DTa, from a DNA vaccine enhances the immune response. Immunol Cell Biol.

[4] Gummow J (2015). A Multiantigenic DNA Vaccine That Induces Broad Hepatitis C Virus-Specific T-Cell Responses in Mice. J Virol.

[5] Grubor-Bauk B (2016). Intradermal delivery of DNA encoding HCV NS3 and perforin elicits robust cell-mediated immunity in mice and pigs. Gene Ther.

[6] Gargett T (2014). Increase in DNA vaccine efficacy by virosome delivery and co-expression of a cytolytic protein. Clin Transl Immunology.

[7] Brennan AJ (2011). Protection from endogenous perforin: glycans and the C terminus regulate exocytic trafficking in cytotoxic lymphocytes. Immunity.

[8] Kono H, Rock KL (2008). How dying cells alert the immune system to danger. Nat Rev Immunol.

[9] Joffre OP, Segura E, Savina A, Amigorena S (2012). Cross-presentation by dendritic cells. Nat Rev Immunol.

[10] Melcher A (1998). Tumor immunogenicity is determined by the mechanism of cell death via induction of heat shock protein expression. Nat Med.

[11] Gallucci S, Lolkema M, Matzinger P (1999). Natural adjuvants: endogenous activators of dendritic cells. Nat Med.

[12] Sauter B (2000). Consequences of cell death: exposure to necrotic tumor cells, but not primary tissue cells or apoptotic cells, induces the maturation of immunostimulatory dendritic cells. J Exp Med.

[13] Chan FK, Moriwaki K, De Rosa MJ (2013). Detection of necrosis by release of lactate dehydrogenase activity. Methods Mol Biol.

[14] Voskoboinik I (2005). Calcium-dependent plasma membrane binding and cell lysis by perforin are mediated through its C2 domain: A critical role for aspartate residues 429, 435, 483, and 485 but not 491. J Biol Chem.

[15] den Haan JM, Lehar SM, Bevan MJ (2000). CD8(+) but not CD8(−) dendritic cells cross-prime cytotoxic T cells *in vivo*. J Exp Med.

[16] Hovav AH (2007). Duration of antigen expression *in vivo* following DNA immunization modifies the magnitude, contraction, and secondary responses of CD8+ T lymphocytes. J Immunol.

[17] Quah, B. J. & Parish, C. R. The use of carboxyfluorescein diacetate succinimidyl ester (CFSE) to monitor lymphocyte proliferation. *J Vis Exp* (2010). 10.3791/2259 PMC3185625 20972413

[18] Romani N (2012). Targeting skin dendritic cells to improve intradermal vaccination. Curr Top Microbiol Immunol.

[19] Akbari O (1999). DNA vaccination: transfection and activation of dendritic cells as key events for immunity. J Exp Med.

[20] Porgador A (1998). Predominant role for directly transfected dendritic cells in antigen presentation to CD8+ T cells after gene gun immunization. J Exp Med.

[21] Elnekave M (2010). Directly transfected langerin+ dermal dendritic cells potentiate CD8+ T cell responses following intradermal plasmid DNA immunization. J Immunol.

[22] Corr M, von Damm A, Lee DJ, Tighe H (1999). *In vivo* priming by DNA injection occurs predominantly by antigen transfer. J Immunol.

[23] Cho JH, Youn JW, Sung YC (2001). Cross-priming as a predominant mechanism for inducing CD8(+) T cell responses in gene gun DNA immunization. J Immunol.

[24] Zanetti M, Castiglioni P, Rizzi M, Wheeler M, Gerloni M (2004). B lymphocytes as antigen-presenting cell-based genetic vaccines. Immunol Rev.

[25] Colluru VT, McNeel DG (2016). B lymphocytes as direct antigen-presenting cells for anti-tumor DNA vaccines. Oncotarget.

[26] Bursch LS, Rich BE, Hogquist KA (2009). Langerhans cells are not required for the CD8 T cell response to epidermal self-antigens. J Immunol.

[27] Kaplan DH (2010). *In vivo* function of Langerhans cells and dermal dendritic cells. Trends Immunol.

[28] Hildner K (2008). Batf3 deficiency reveals a critical role for CD8alpha+ dendritic cells in cytotoxic T cell immunity. Science.

[29] Bedoui S (2009). Cross-presentation of viral and self antigens by skin-derived CD103+ dendritic cells. Nat Immunol.

[30] Kim TJ (2014). Clearance of persistent HPV infection and cervical lesion by therapeutic DNA vaccine in CIN3 patients. Nat Commun.

[31] Larocca RA (2016). Vaccine protection against Zika virus from Brazil. Nature.

[32] Abbink P (2016). Protective efficacy of multiple vaccine platforms against Zika virus challenge in rhesus monkeys. Science.

[33] Meurens F, Summerfield A, Nauwynck H, Saif L, Gerdts V (2012). The pig: a model for human infectious diseases. Trends Microbiol.

[34] Birnboim HC (1983). A rapid alkaline extraction method for the isolation of plasmid DNA. Methods Enzymol.

[35] Holmes, K., Lantz, L. M., Fowlkes, B. J., Schmid, I. & Giorgi, J. V. Preparation of cells and reagents for flow cytometry. *Curr Protoc Immunol* Chapter 5, Unit 5 3 (2001). 10.1002/0471142735.im0503s44 18432799

